# Enhancer Dysfunction in 3D Genome and Disease

**DOI:** 10.3390/cells8101281

**Published:** 2019-10-19

**Authors:** Ji-Han Xia, Gong-Hong Wei

**Affiliations:** Biocenter Oulu, Faculty of Biochemistry and Molecular Medicine, University of Oulu, 90014 Oulu, Finland

**Keywords:** enhancer chromatin, epigenetic marks, chromatin looping and 3D genome, gene transcription, cancer, cancer risk variants, GWAS, allele-specific chromatin binding

## Abstract

Spatiotemporal patterns of gene expression depend on enhancer elements and other factors during individual development and disease progression. The rapid progress of high-throughput techniques has led to well-defined enhancer chromatin properties. Various genome-wide methods have revealed a large number of enhancers and the discovery of three-dimensional (3D) genome architecture showing the distant interacting mechanisms of enhancers that loop to target gene promoters. Whole genome sequencing projects directed at cancer have led to the discovery of substantial enhancer dysfunction in misregulating gene expression and in tumor initiation and progression. Results from genome-wide association studies (GWAS) combined with functional genomics analyses have elucidated the functional impacts of many cancer risk-associated variants that are enriched within the enhancer regions of chromatin. Risk variants dysregulate the expression of enhancer variant-associated genes via 3D genomic interactions. Moreover, these enhancer variants often alter the chromatin binding affinity for cancer-relevant transcription factors, which in turn leads to aberrant expression of the genes associated with cancer susceptibility. In this review, we investigate the extent to which these genetic regulatory circuits affect cancer predisposition and how the recent development of genome-editing methods have enabled the determination of the impacts of genomic variation and alteration on cancer phenotype, which will eventually lead to better management plans and treatment responses to human cancer in the clinic.

## 1. Introduction

Various studies have shown that most regulatory driver genetic variants are located in the noncoding region of the genome. Various approaches, such as comparative and evolutionary genomics as well as biochemical methods, have enabled the identification of the functional regulatory elements and the modes of their interactions [1,2,3,4]. The role of the noncoding part of the genome was not extensively explored initially after the Human Genome Project, but advancements in next generation sequencing (NGS) technologies have opened doors to understanding noncoding genome by providing large sets of multiomic data. Noncoding single nucleotide polymorphisms (SNPs) have been found to be associated with various common disorders, and therefore, defining their locations and functions has become urgent. To identify the functional and regulatory roles of the noncoding part of the human genome, the National Human Genome Research Institute (NHGRI) launched “The Encyclopedia of DNA Element (ENCODE) Project,” which revealed that approximately 80% of the genome is functionally involved in biological activities [5,6]. Later, a similar project, modENCODE, was launched for the same purposes with respect to fruit fly and worm genomes [7].

**Figure 1:**
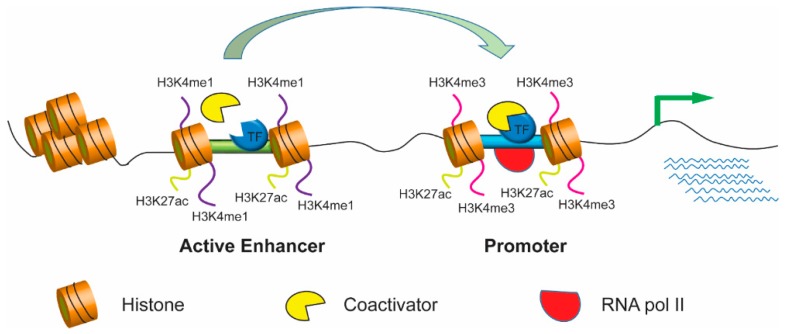
Active enhancers promote high-level gene expression. Epigenetic marks for active enhancers usually include H3K4me1 (monomethylation of H3 lysine 4) and H3K27ac whereas the trimethylation of histone H3 lysine 4 (H3K4me3) is often enriched at gene promoters. The active enhancers regulate gene transcription through chromatin looping with the promoters of target genes. Thus, looping formation eventually contributes to the recruitment of transcription factors, coactivators, and RNA polymerase, promoting high levels of target gene expressions.

Most genome-wide SNPs have reportedly been found in noncoding regions containing enhancers, which are cis-regulatory elements first identified in the SV40 virus genome, that can act with promoters over long distances [8,9]. Enhancer location is highly variable—it can be upstream or downstream or within the target gene. The activity of target genes can be modulated by enhancers independent of their location. Enhancer activity can be attenuated or blocked by other cis-regulatory elements called silencers and insulators, respectively [10]. Enhancers usually contain multiple binding sites for trans-acting proteins called transcription factors, which initiate the transcription process by recruiting various coactivators in coordination with RNA polymerase II and the transcription initiation complex [11,12,13]. Binding of transcription factors also enables modification of chromatin [11,14,15,16]. Active enhancers are completely devoid of nucleosomes; therefore, they are accessible to the transcription factors that bind to them. However, the vicinity of enhancer regions containing nucleosomes has unique histone monomethylation and acetylation signatures [6,17,18]. Active enhancer chromatins are marked by H3 lysine 4 monomethylation (H3K4me1) and H3 lysine 27 acetylation (H3K27ac) but not histone H3 lysine 4 trimethylation (H3K4me3) [14]. Furthermore, an active enhancer can interact with the promoter of target genes, which contributes to the recruitment of transcription factors and coactivators and the initiation of RNA polymerase dependent transcription, leading to gene expression (Figure 1).

Genome-wide association study (GWAS) approach can be used to identify disease-associated SNPs but not explain their functional roles or molecular mechanisms. However, many recent studies have shown that the SNPs in enhancer regions affect the normal gene regulation process. For example, rs339331 alters the binding affinity of the transcription factor HOXB13, which induces the expression level of the 6q22 locus gene *RFX6* in prostate cancer cells [19]. Various other projects, like 1000 GENOME and individual genome projects, have also provided large data sets of genetic variations in both coding and noncoding DNA that are associated with phenotypic divergence and disease susceptibility in various ethnic groups [20,21,22,23]. NGS technologies have led to the identification of millions of enhancers, though the functions of the majority of them remain unknown, the data can be applied to interpret causation of GWAS-discovered genomic variants.

This review emphasizes the discovery of cis-regulatory elements, namely enhancers, with elaboration on the efficacy of different methods used to study them and suggestions for improving these techniques. We also discuss enhancer dysfunction and its role in disease progression, and how genome editing strategies can be applied to remedy genetic variations that lead to disease susceptibility for potential application in clinical therapeutics.

## 2. Progress in Genome-Wide Dissection of the Cis-Regulatory Code

### 2.1. New Knowledge of the Cis-Regulatory Code

A type of proteins, namely transcription factors that bind to specific DNA sequences, determine the information encoded in enhancer sequences to increase the transcription of specific genes. Transcription factor DNA-binding sites are generally 6–20 bp long. Various computational methods, such as position weight matrix (PWM) programing, have been established to define consensus binding motifs for specific transcription factors. Furthermore, many computer-aided or manually curated databases—such as TRANSFAC [24], JASPAR [25], and UniPROBE [26]—provide information acquired from different studies on the preferential binding of transcription factors. However, these transcription factor DNA-binding motifs do not guarantee the same binding affinity of given transcription factors to active enhancers.

**Figure 2:**
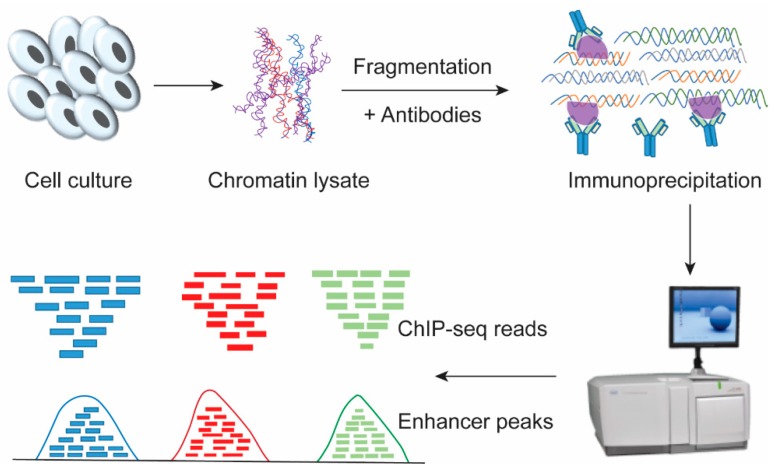
Genome-wide identification of cis-regulatory drivers, the enhancers. The cells were cross-linked with formaldehyde, and glycine was added to stop the reaction. Cell pellets were collected and suspended to isolate the nuclei. Chromatin was prepared by sonication into certain size, and the fragments were incubated with antibodies against target proteins. Then, extraction buffer was added to extract and purify the DNA from the complexes. The target DNA fragments were enriched and sequenced by using ChIP-seq in combination with bioinformatics analysis. According to the called ChIP-seq peaks, the enhancer elements in the chromatin can be identified.

In recent years, the revolution in technology, especially high-throughput sequencing, has greatly increased our knowledge by identifying a large number of cis-regulatory elements, although many of them still need to be validated. Genome-wide evolution-based methods were used for finding enhancers that are highly conserved across species. Chromatin immunoprecipitation followed by massively parallel sequencing (ChIP-seq; Figure 2) is the most frequently used technology for identifying enhancer sequences across the genome, but it does not provide information on the regulatory roles or regulated target genes. The ChIP-seq method involves the cross-linking of cells by formaldehyde such that the physiological transcription factors-DNA interactions can be chemically fixed, and then, the chromatin is sheared into 300~1000 bp by sonication or enzyme, which enables specific recognition of antibodies with transcription factors-chromatin complexes [27,28,29,30]. The immunoprecipitated protein-DNA complexes were reversely crosslinked and the purified ChIP DNA was sequenced and analyzed. Another variant of ChIP-seq, called ChIP-exo, gives results at higher resolution and involves an additional step for exonuclease digestion, which cuts the DNA into fragments [31].

It is now well known that active enhancers are in regions with depleted nucleosomes enabling transcription factors and other mediators to access genome DNA. These nucleosome-depleted open chromatin regions and the nucleosome positioning across the genome can be detected by the endonuclease activity of DNase I or micrococcal nuclease (MNase) followed by deep sequencing (DNase-seq and MNase-seq, respectively) [32,33,34,35]. Alternatively, FAIRE-seq (formaldehyde-assisted isolation of regulatory elements) and ATAC-seq provide information about the nucleosome-free region by cross-linking cells and using respective tagged transposon elements followed by deep sequencing [36,37,38]. However, similar to DNase-seq and MNase-seq, ATAC-seq also shows preferential cleavage towards specific sequences [37,39,40]. 

Since its discovery, the genetic code was presumed to be exclusive for defining protein formation, but a recent study explains that the genetic code has two functions: First, protein formation and, second, gene regulation through transcription factor DNA-binding specificity [41,42]. Various approaches have been used to understand the binding specificity of DNA and transcription factors. In vivo methods that measure transcription factor-DNA binding specificity are ChIP-on-chip, ChIP-seq, and more recently, ChIP-exo as described above. However, various factors influence the binding between transcription factors and chromatin DNA when these in vivo methods are used. In contrast, in vitro binding experiments are the best approaches for measuring transcription factor-DNA interaction specificity and affinity. The most widely used in vitro approach is protein binding microarray (PBM). PBM involves the simple hybridization of the tagged transcription factor with double-stranded DNA, which subsequently produces a fluorescence signal that reveals the binding strength [43]. A newer technology, high-throughput SELEX (Systematic Evolution of Ligands by EXponential Enrichment), is also used to characterize the relative binding specificities of DNA sequences to transcription factors [44,45]. Moreover, SELEX gives more accurate results compared to those generated through the use of PBMs.

### 2.2. Genome-Wide Methods to Study Regulatory Interactions

**Figure 3:**
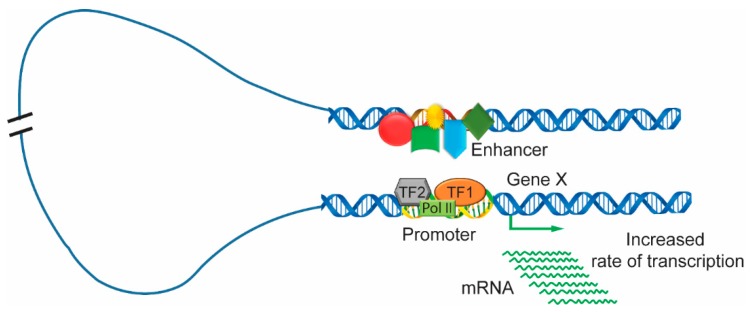
Looping between clusters of activators, mediators and transcription factors in enhancer and promoter regions. The enhancer region of chromatin is bound by the transcriptional complexes including various activators, mediators and transcription factors, which form enhancer clusters. The enhancer interacts with the promoter region, which combines with transcription factors and RNA polymerase to increase the rate of gene transcription.

The evolution of ChIP-seq has advanced the genome-wide identification of enhancers, which are most often directed by transcription factor binding. The recently developed chromosome conformation capture (3C) and the 3C-derived methods provide knowledge regarding the spatial proximity and physical interaction between enhancers and promoters [46,47]. 3C technology is most widely used to map the long-distance interactions between promoters and enhancers. In addition, its derivatives, circular chromosome conformation capture (4C) and circular chromosome conformation capture carbon copy (5C), respectively, are used to examine the interaction at one genomic location with many other genomic locations throughout the whole genome [48,49]. Notably, Hi-C is also based on the same strategies as those of 3C, with a slight adjustment: biotin-labeled nucleotides are added at restriction ends to study the long-range enhancer and promoter communication in a genome-wide fashion [50,51,52]. All these assays involve the cross-linking of cells with formaldehyde, shearing and ligation with genome DNA to generate a chimeric DNA that has close physical contact with a long distance location [48] and to identify enhancers through bioinformatics analysis (Figure 2 and Figure 3). Furthermore, ChIA-PET, which is a combination of 3C and chromatin immunoprecipitation, is based on paired end tagging with next-generation sequencing and enables the analysis of chromatin interaction at sites bound by specific DNA-binding proteins, which is useful for determining the long-distance interactions between promoters and enhancers [53]. 3C methods provide information about the spatial arrangement of the genome and the physical interactions between enhancers and promoters (three-dimensional (3D) genome), but they do not provide any information about the regulatory interactions of transcription factors. Furthermore, neither 3C nor its derivatives, which map regulatory interactions across the genome, provide information on all the physical interactions that have some regulatory roles [54,55]. Therefore, we can expect that continued advancement in technologies will improve the resolution of these mechanisms and our understanding of regulatory interactions mediated through certain transcriptional complexes.

## 3. Importance of Enhancers as cis-Regulatory Drivers

### 3.1. Lineage-Specific Factors Mediate Long-Distance Interactions with Enhancers

Enhancers increase transcription independent of their orientation, position, or distance to a promoter and establish spatiotemporal and cell-type-specific patterns of gene expression. Although the first enhancer was discovered in mammalian cells, our knowledge of enhancers has been significantly increased by using *Drosophila* embryos. The enhancer of the *cut* (ct) gene regulates expression in *Drosophila* wing imaginal disc cells and is situated 81.5 kb upstream from its transcription start site [56]. However, the limb bud enhancer for the mouse *Sonic hedgehog* (*Shh*) gene is located in the intron of another gene more than 1 Mb from the *Shh* gene promoter [57,58]. The ENCODE project combines epigenomic profiling technologies and considers enhancer-associated chromatin features to annotate enhancers throughout the whole genome [6,59]. This project also indicates that enhancers are the most dynamic parts of the genome and has identified a myriad of putative enhancers in different cell lines, indicating the combinatorial complexity of gene expression during the developmental process [60,61].

It is well understood that the activation of gene transcription proceeds through many steps. Enhancers bound to transcription factors undergo many dynamic and progressive changes during different stages of development and expose active positions at certain times. The loop mechanism is a well-recognized model for the interaction of distal enhancers; the DNA is looped in such a way that the enhancer comes in very close proximity to the promoter [62,63,64,65,66]. The enhancer region is usually bound with many clusters of activators, mediators, and transcription factors. It interacts with the gene promoter that is in association with transcription factors and RNA polymerase. Then, the enhancer can initiate transcription and upregulate the levels of gene expression (Figure 3). In addition, another model of enhancer function, termed the ‘tracking’ or ‘scanning’ mechanism, is based on promoter and enhancer interactions caused by the free diffusion or the facilitated movement of the enhancer along chromatin fibers as it searches for a promoter with which to interact [63,67]. Later, another model, termed the ‘linking’ or ‘oozing’ model, was proposed, and it is based on a complex nucleated at the enhancer and polymerized bidirectionally along the chromatin fiber until it reaches the promoter [62,68]. For example, in mature blood cells, dimerized LIM domain binding protein (LDB1) in a complex with LMO2, GATA1, and FOG1 were required for the β-globin locus control region to loop to the globulin gene [69,70,71]. The looping model of the enhancer-promoter interaction is greatly supported by 3C and its high-throughput derivatives. The 3C approach indicates the frequency of the physical interactions between the genomic location by using cross-linked cells followed by restriction digestion and DNA ligation and, finally, PCR analysis of the product [46,72]. This method provides the interaction strength if both genomic positions are in the same three-dimensional space in the nucleus. Another 3C-derived technology combined with next-generation sequencing maps the interaction of one genomic location to the whole genome and is called circular chromosome conformation capture (4C) [73].

### 3.2. Spatiotemporal Organization of Enhancers in the Nucleus

3C and the 3C-derived approaches have improved our perception of regulatory genome context and 3D chromatin structures. The mammalian genome is arranged in a series of conserved topologically associated domains (TADs) with dozens of genes and enhancers, as suggested by 3C [54,74,75]. The chromatin loci interactions within TADs are more frequent than those between different TADs. The highly self-interactive TADs are significantly insulated from nearby regions, which is a critical condition for modifying the 3D structures associated with enhancer–promoter interaction and gene expression [76,77]. TADs can be transcriptionally active or inactive during differentiation and development and can differ in size [78,79,80,81]. The super enhancers surrounded by TADs are clusters of typical enhancers that are associated with a large number of mediators, and are found in pluripotent cells and encode regulators of cell identity and disease [76,82].

Chromatin dynamics and 3D structure play critical roles in determining cellular fate, differentiation, pluripotency, identity, and plasticity, guaranteeing that each cell performs the proper function in every tissue and organ. CTCF sites are enriched near the margins of TADs, reflecting the role of these TADs in distant interactions, but how the margins differ from each other remains unknown. The functional relevance of TADs in distant interactions was verified by the transcription misregulation due to the deletion of the TAD margin in the Xist locus [83]. CTCF binding and cohesion play different roles at different levels in distant interactions. Reducing CTCF binding in HEK293T cells increased TAD interactions with few changes in gene expression, whereas reduced cohesion had no role in TAD interactions in thymocytes, indicating the functional role of CTCF during long-distance interactions [84,85,86,87]. Cohesin loops regulate the interaction between gene enhancers and promoters, which suppress aberrant enhancer and promoter contacts [10]. Ke et al. described the dynamics of 3D chromatin structures during mouse development from gamete to early embryo, showing the process of genome structure establishment from an obscure structure in the zygote to a mature 3D structure [88]. TADs, which are genomic regulatory units, have internal stability and are affected by structural genomic variations acquired during development and disease processes.

## 4. Effect of DNA Methylation on Enhancer Activity

Genome-wide studies support the view that DNA methylation has a negative impact on enhancer activity [89,90,91,92]. There is a significant association between the enhancer epigenetic signature, transcription factor binding and enhancer activity at the distal promoter region in neural progenitor cells and embryonic stem cells [55,93,94,95]. A recent study revealed that gene activity changes because of altered gene methylation in medulloblastoma, and are similar to the activity differences between normal cells and other types of cancerous cells. This study also reported that 1000 genes are methylated at low levels in medulloblastoma cells compared to the levels of their healthy counterparts [96]. In addition, a study proposed the term vestigial enhancers to define early enhancers, which are methylated in adult tissue with repressive histone methylation (H3K27me3) [97,98,99]. Furthermore, another study found that 5mC oxidation and hypomethylation are essential mediators of enhanced Tet2 activity, as determined during the epigenomic profiling of a Tet deletion.

## 5. Enhancer-Produced Enhancer RNA (eRNA)

Active enhancers are bound by several transcription factors with RNA pol II to produce RNA called enhancer RNA (eRNA) [100,101], which can be short, bidirectional and non-polyadenylated or long, unidirectional, and polyadenylated [102]. However, the function and mechanism of eRNA directionality require proper investigation. Most recently, the FANTOM5 project and results from recent studies showed that short bidirectional RNA is a signature for active enhancers and participates in looping for long-distance interactions [103]. The FANTOM5 project provides an online catalog of 43,011 active enhancers based on the CAGE library, which encompasses 432 primary cell lines, 135 tissue types, and 241 cell lines from human samples [103]. This project revealed a few untranscribed enhancers and chromatin features that affect transcription, suggesting that many chromatin-derived enhancers do not have regulatory activity in certain cells but may be active in other types of cells in the same lineage [103,104,105]. The function of eRNA is still a topic for further research; however, a recent study has reported the important role of eRNA in elongation. Specifically, eRNA acts as a decoy for a negative elongation factor, which helps transition paused RNA pol II into the productive elongation stage in the early phase of neuron development [106]. The exact function of enhancer RNA is not properly understood. A recent study identified a class of lncRNAs, similar to eRNAs, using human genome annotation data from GENCODE, that participate in gene activation and function similar to enhancers in human cells. Although the specific mechanism of the enhancer-like functions of lncRNAs has not yet been discovered, experimental results of a study showed that lncRNA-facilitated gene expression is orientation independent, mediated by RNA and not cell-specific [107].

## 6. Types of Enhancers—Super Enhancers, Stretch Enhancers, and Shadow Enhancers

There are several types of enhancers with different characteristics. Enhancers are responsible for increasing transcriptional output. Similar to the *Drosophila* HOT (highly occupied target) region [7], clusters of transcription factors with mediator complexes have been found in mammalian pluripotent cells and are called super enhancers. Compared to typical enhancers, super enhancers are bound by more transcription factors, which are larger and capable of dramatically increasing transcriptional activity [82,108,109]. Clusters containing super enhancers and highly transcribed TADs interact simultaneously with multiple partners in the 3D structure of mES cells, which expanded the reporting of cluster-bound SOX2 and the pairwise contacts of the super enhancers. These super enhancers might be the origins of super enhancer-containing TADs [108]. Previously, it was assumed that ES cells control gene expression through transcription factors, including Oct4, Sox2, and Nanog (OSN), but six additional transcription factors have recently been reported to contribute to gene expression in ES cells. The analysis revealed that super enhancers also control cell-type-specific gene expression, which is very sensitive to reduced levels of mediator complexes and transcription factors. In addition, the study provided a catalog of super enhancers in 86 human cells and tissues with different traits and associated diseases, explaining a large collection of GWAS discoveries [110].

The stretch enhancer was revealed during the epigenetic profiling of human pancreatic islets. Computational analysis of type 2 diabetes data revealed a wide region of enhancers that was greater than 3 kb comprising so-called stretch enhancers or extended enhancers. Similar to super enhancers, stretch enhancers also increase gene expression and work in a cell-type-specific manner. In the same study, the data illustrated the role of the stretch enhancer in common human diseases due to enrichment of associated SNP in the same region [111].

The term shadow enhancer is used to define a secondary enhancer of the *Drosophila* genes *sog* and *brk*, which drive gene expression in the neuroectoderm of the embryo. In *Drosophila*, the pattern of gene expression is generally controlled by two enhancers that are called the primary enhancer and the secondary enhancer. The secondary enhancer, which is distant from the target gene, acts with the primary enhancer to regulate gene expression [112].

## 7. Identification of Chromatin Marks for Enhancers

Genome-wide technologies have been used to describe a large number of putative enhancers on the basis of histone modification in ChIP data, suggesting that enhancers display characteristic chromatin marks, which play important roles in transcriptional regulation. The ENCODE project results suggest that enhancer chromatin marks can be identified by the presence of H3 lysine 4 monomethylation (H3K4me1) but the absence of the histone H3 lysine 4 trimethylation (H3K4me3) [5,7]. H3 lysine 27 acetylation (H3K27ac) is also used as a signature for identifying an enhancer [6,113]. Many studies have indicated the significant presence of H3K4me3 at promoter sites and the reduced presence of H3K4me1. A study presented a model for the identification of active enhancers and inactive enhancers in embryonic stem cells and illustrated that H3K4me1 in the absence of acetylated H3K27 was indicative of an inactive enhancer or a poised enhancer. It is possible that the active enhancer is sensitive to the presence of both H3K27ac and H3K4me1 [114,115]. The active enhancer mark H3K27ac was first observed in *Drosophila* in CBP and was later reported in mammals in the CBP paralogs P300 and CBP. P300 and CBP are both involved in a coactivator complex and acetylate a number of histones. Genome-wide studies have shown that many enhancers lose their signature mark during the differentiation process of embryonic stem cells and this loss is necessary for enhancer decommissioning [88,116].

## 8. Enhancer Dysfunction and Human Disease

**Figure 4:**
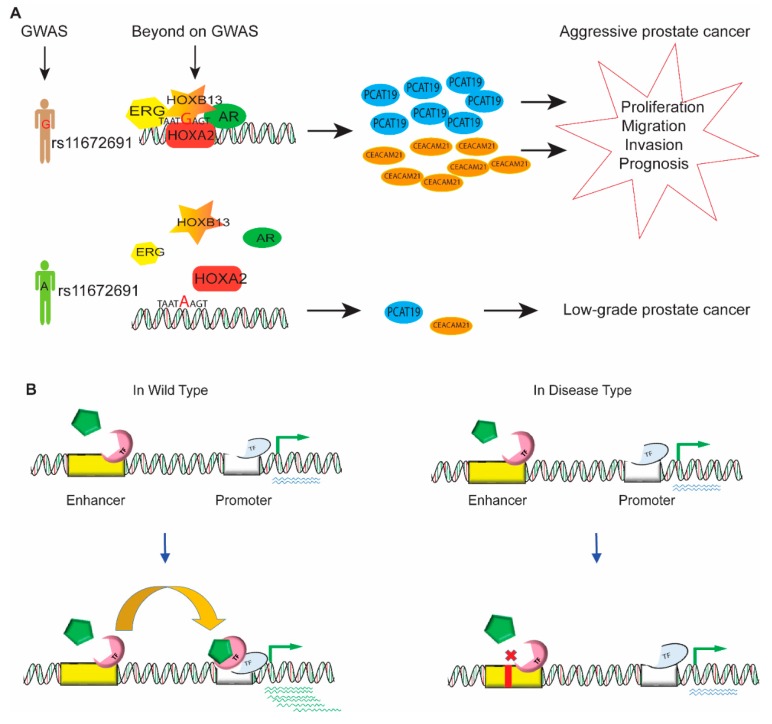
Enhancers in gene regulation and disease. (**A**) The aggressive G allele of rs11672691 enhances the binding of transcription factor HOXA2, thus increasing the expression level of the plausible candidate genes PCAT19 and CEACAM21, which results in prostate cancer cell growth and progression. (**B**) In wild-type cells, the driver of transcription factor binding with other factors in the enhancer region contributes to promoter interaction with the promoter of target genes to upregulate gene expression. However, in disease conditions, genetic variation and chromosomal aberrations affect the binding affinity of clusters of activators, mediators, and transcription factors, thereby dysregulating the expression of target genes.

Successful completion of various human genome projects revealed that the human genome largely consists of noncoding DNA, which has no role in protein expression, but the preliminary result of the ENCODE project changed this presumption by defining the role of noncoding DNA in most (up to 80%) of the biochemical and regulatory activity of the genome. Genome-wide association studies and epigenomic profiling coupled with high-throughput sequencing have been used to identify a large number of genetic variations and chromosomal aberrations, which are risk factors for disease in the human genome, especially in cancer initiation and tumor progression [117]. However, it is still very difficult to determine the functional role of the genetic variations in noncoding DNA. These noncoding risk variants usually affect the cis-regulation of gene expression that results in the phenotypic variations in complex traits and cancer. For example, the risk SNP rs11672691, which is associated with aggressive prostate cancer, mediated the interaction of promoters and enhancers and regulated the expression of lncRNA *PCAT19* [118,119]. Gao et al. revealed that the aggressive G allele of rs11672691 enhanced the binding of transcription factor HOXA2 and elevated the levels of the plausible candidate genes *PCAT19* and *CEACAM21*, which are implicated in prostate cancer cell growth and progression (Figure 4A). A wide range of acting enhancers in the noncoding region of DNA participates in various regulatory activities of gene regulation and developmental processes. This part of the review discusses enhancer dysfunction due to genetic variations and chromosomal aberrations and addresses the ways these variations cause disease. Genetic variations and chromosomal aberrations affect the binding affinity of clusters of activators, mediators, and transcription factors, thereby dysregulating the expression of target genes and causing human disease (Figure 4B).

Inappropriate epigenetic modifications and/or genetic variants in the enhancer region greatly contribute to humancarcinogenesis. The analysis of innumerable epigenetic abnormalities and somatic mutations in chromatin regulatory factors showed that epigenetic disruption is a major hallmark of cancer progression. Accumulating evidence has revealed that aberrant enhancer and super enhancer clusters playing important roles in the activation of oncogenes and the dysregulation of tumor suppressor genes [110]. Enhancers are more likely to be altered than promoters in many human cancer cell lines and in different patients, resulting in normal tissue malignancy to primary tumors and even metastases [120]. For example, Chen et al. analyzed thousands of tumor samples across 33 cancer types and observed global enhancer activation in most cancers. They further discovered interactions of causal enhancer-genes and identified an enhancer of PD-L1, which is a major immunotherapy target, suggesting that enhancers have potential clinical applications [67]. Corces et al. investigated genetic risk loci of cancer that are active DNA regulatory elements and identified regulatory interactions involved in cancer immune evasion and pinpointed noncoding mutations, which affected enhancer activation and patient survival in 410 tumor samples representing 23 cancer types in TCGA (The Cancer Genome Atlas) [38]. Moreover, the driver and enhancer of causal gene contribute to cancer progression. For example, Bahr et al. showed that the enhancer of the *MYC* gene is essential for regulating the expression of *MYC* in hematopoietic malignancies [121]. Roe et al. reported that FOXA1 is a driver of enhancer activation, rendering pancreatic ductal adenocarcinoma cells more invasive and metastatic [17]. In addition, aberrant enhancer or variation involve in the oncogenesis. For example, Xiong et al. reported that aberrant enhancer hypomethylation in hepatocellular carcinomas contributes to hepatocarcinogenesis through whole genome transcriptional reprogramming [90]. A risk variant in a noncoding distal enhancer element regulates the expression of SNCA (α-synuclein), a key gene involved in the pathogenesis of Parkinson’s disease [9].

## 9. Enhancer Hijacking

Recently, studies have shown that enhancer hijacking plays a significant role in medulloblastoma and oncogenesis [122]. Previously, this kind of activity had been reported in the immunoglobulin gene due to the translocation of MYC and BCL2 in the active enhancer region. Medulloblastoma comprises four groups, and two groups, group 3 and group 4, showed an important role in most cases of pediatric malignancy [121,123]. The results from this study indicated that genomic rearrangements are responsible for activating growth factor-independent 1 family proto-oncogenes GFI1 and GFI1B, which are in medulloblastoma group 3 and group 4. Earlier reports emphasized the role of somatic MYC and MYCN gene amplification as a prevalent driver of medulloblastoma, but recent analyses of the whole genome sequencing data for medulloblastoma, in which all chromosomal rearrangements were considered, led to the identification of a novel region with highly disparate structural variant classes—i.e., focal deletion, tandem duplication, and inversion—in chromosome 9q34.13. These highly disparate genomic rearrangements led to the oncogene activation of GFI1 and GFI1B, and results from the epigenomic profiling of the region suggested that the active enhancer mark was that of enhancer hijacking [124,125]. Haller et al. reported that the rearrangement of an enhancer upregulated the expression of NR4A3 (nuclear receptor subfamily 4 group A member 3), which increased the expression of NR4A3 target genes and stimulated cell proliferation, suggesting the importance of enhancer hijacking in salivary gland acinic cell carcinoma [124]. Structure variants disturb TAD boundaries, which are likely caused by enhancer hijacking, exposing enhancers to new TSS (transcription start sites) in both human cancer and other disease [125].

## 10. Super Enhancer Variations

Sequence variation in the super enhancer domain can result in specific diseases. Many diseases are reportedly associated with SNPs in super enhancer regions in relevant cell types. 76 SNPs have been reported for type-1 diabetes, 13 of which associate the super enhancer region. Furthermore, systemic lupus erythematosus also is associated with 22 SNPs found the super enhancer regions of 16 genes [110]. The super enhancer of *MYC* is essential for the leukemic stem cell hierarchy and hematopoiesis in humans and mice, and it has multiple enhancer modules that recruit a number of transcription factors, including MYB and RUNX. Deletion of this super enhancer causes a complete loss of *MYC* expression in hematopoiesis cells and an accumulation of multipotent progenitors [121].

## 11. CRISPR/Cas9 System for Genome Editing

Genome editing was hampered by lack of an efficient tool that can break double-stranded DNA at a designed place. CRISPR (clustered regularly interspaced short palindromic repeats)/Cas9 was originally identified in the archaeal adaptive immune system of bacteria that was based on RNA-guided degradation of foreign nucleic acid. The CRISPR/Cas9 system has proven efficiency in many different organisms, including humans [126]. CRISPR/Cas9 is more cost-effective and efficient than the earlier genomic editing methods, i.e., zinc finger nuclease and TALENS (transcription activator-like effector nucleases). This technique is widely used for genome editing. A study compared the CRISPR/Cas9 system and TALENS, and the results indicated that the efficiency of the CRISPR/Cas9 system is superior to that of TALENS [127]. The CRISPR/Cas9 system of genome editing is very useful in manipulating DNA sequences and correcting them at the molecular level. We can manipulate the enhancer DNA sequence by creating the insertion or deletion to determine the influence on gene activity [128]. Recently, a study used CRISPR/Cas 9 to regulate gene expression by targeting enhancers in iPS cells, and the results suggested preferential activity of activators inside the enhancer region [127]. The study has indicated that a CRISPR/Cas9-mediated large genomic deletion can be created easily in mammalian cells, suggesting that a deletion strategy could be useful for generating mutations in the noncoding region [128,129,130]. Furthermore, a large library of gRNAs that is useful for altering or regulating genes of interest is expected to be helpful in understanding genetic mechanisms. The high efficiency of the CRISPR/Cas9 system has revolutionized biomedical research and genomic regulation. For example, Pan et al. used a CRISPR-Cas9 genome screen to explore the molecular mechanism of tumor cell resistance to cytotoxic T cells and discovered that the tumors were resistant to immunotherapy when *PBRM1* was inactivated [129]. Matharu et al. reported that CRISPR-mediated activation of an enhancer or promoter of *Sim1* in heterozygous mice rescued the obesity phenotype [131]. In addition, the CRISPR/Cas9 is useful technology for cancer risk variants research. For example, Soldner et al. reported a novel method to functionally identify the cis-regulatory genetic risk variants in gene expression by combining genome epigenetic information with CRISPR/Cas9 genome editing in human pluripotent stem cells [9]. Ping et al. and Junjie et al. applied the CRISPR/Cas9 system to clarify the molecular mechanisms of how the risk SNP rs11672691 affects enhancer activity and causes progression of aggressive PCa [118,119]. The CRISPR system is expected to be helpful in targeting enhancers to understand how they modulate the transcription of genes [126,131,132,133].

## 12. Conclusions and Perceptive

**Figure 5:**
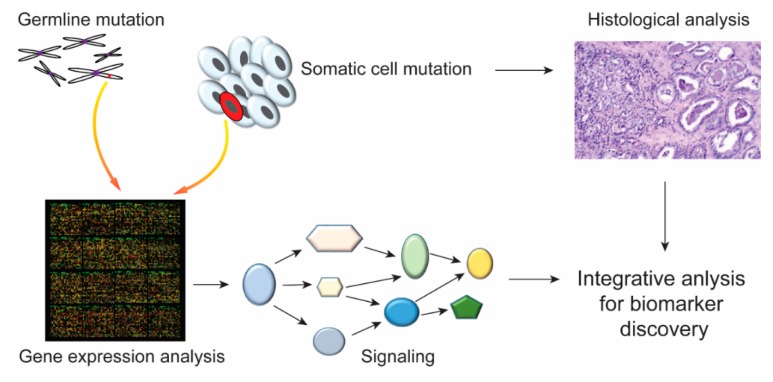
Integrated analysis of germline and somatic mutations. The mutation in germline and somatic cells can be tested by high-throughput sequencing techniques at the genetic or transcript level. Integrated analyses of disease-related gene expression or pathways combined with histological data might contribute to the identification of biomarkers for disease diagnoses and treatment.

Gene regulation in eukaryotes is a complex process of recruiting various transcription factors, activators and repressors to the cis-regulatory module at different stages of development and disease. Most critical regulation of gene expression occurs at the transcription level. Therefore, it is important to identify the cis-regulatory sequences and their functional activity. It is essential to find biomarkers for disease diagnosis and treatment. The mutations in germline and somatic cells were analyzed by high-throughput sequencing techniques at the genome or transcript level. Then, integrative analysis of disease-related genes or pathways combined with histological data might contribute to the identification of biomarkers for clinical treatment (Figure 5). Somatic mutation of a noncoding region may lead to an oncogenic super enhancer. Moreover, the noncoding region activates nearby target genes, which may also encode for proteins that function as tumor suppressors [52]. In this review, we have discussed the genome-wide methods used for enhancer identification, their mode of action, unique chromatin features, and histone modifications. We have also looked into enhancer dysfunction due to chromosomal rearrangements and a genome-editing strategy for remedying the genetic variation by insertion or deletion.

Advancements in genome-wide methods for the identification of cis-regulatory elements, i.e., enhancers, have revolutionized the field of regulatory genomics. However, ChIP-seq methods for recognizing cis-regulatory elements also have biased towards specific DNA sequences [35]. Genome-wide methods combined with high-throughput tools have provided a tremendous number of putative enhancers, which are slowly being validated for their regulatory activity. The biggest challenge is determining the appropriate functional annotation of the identified enhancer and then linking the enhancer to the target gene. After finding the target gene, it will be possible to identify the affected pathway.

Recently, genome-editing methods have yielded efficient results for various organisms. With the help of CRISPR/Cas9 and TALENs, both transcription factors [134] and cofactors [135] can be recruited at a predetermined locus. Furthermore, a recent study indicated that CRISPR/Cas9 insertions or deletions can be very effective in directly targeting of enhancers. These genome-editing methods require proper optimization and are expected to be particularly useful in the future.

Considering all these recommendations, future work should focus more on improving technology to make tools more robust and powerful for functional research on enhancers. Furthermore, genome-wide data should be integrated into analysis in a more meaningful way that may provide new breakthroughs in enhancer identification, which will be helpful overall in transcriptional regulation. An optimized CRISPR/Cas9 system will be more effective in targeting enhancers and modifying the desired location. Increasing evidence has revealed that the enhancer region might play a critical role as a key therapeutic target in clinical applications for human cancers and other diseases.
